# Bromelain inhibits the ability of colorectal cancer cells to proliferate via activation of ROS production and autophagy

**DOI:** 10.1371/journal.pone.0210274

**Published:** 2019-01-18

**Authors:** Tung-Cheng Chang, Po-Li Wei, Precious Takondwa Makondi, Wei-Ting Chen, Chien-Yu Huang, Yu-Jia Chang

**Affiliations:** 1 Graduate Institute of Clinical Medicine, College of Medicine, Taipei Medical University, Taipei, Taiwan; 2 Department of Surgery, College of Medicine, Taipei Medical University, Taipei, Taiwan; 3 Division of General Surgery, Department of Surgery, Shuang Ho Hospital, Taipei Medical University, New Taipei City, Taiwan; 4 Division of Colorectal Surgery, Department of Surgery, Taipei Medical University Hospital, Taipei Medical University, Taipei, Taiwan; 5 Cancer Research Center and Translational Laboratory, Department of Medical Research, Taipei Medical University Hospital, Taipei Medical University, Taipei, Taiwan; 6 Graduate Institute of Cancer Biology and Drug Discovery, Taipei Medical University, Taipei, Taiwan; 7 International PhD Program in Medicine, Taipei Medical University, Taipei, Taiwan; University of South Alabama Mitchell Cancer Institute, UNITED STATES

## Abstract

Advanced colorectal cancer (CRC) survival rates are still low despite advances in cytotoxic and targeted therapies. The development of new effective or alternative therapies is therefore urgently needed. Bromelain, an extract of pineapple, was shown to have anticancer effects, but its mechanisms in CRC have not been fully explored. Therefore, the roles of bromelain in CRC progression were investigated using different CRC cell lines, a zebrafish model, and a xenograft mouse model. The anticancer mechanisms were explored by assessing the role of bromelain in inducing reactive oxygen species (ROS), superoxide, autophagosomes, and lysosomes. The role of bromelain in the induction of apoptosis was also assessed. It was found that bromelain inhibited CRC cell growth in cell lines and tumor growth in the zebrafish and xenograft mouse models. It also induced high levels of ROS and superoxide, plus autophagosome and lysosome formation. High levels of apoptosis were also induced, which were associated with elevated amounts of apoptotic proteins like apoptotic induction factor, Endo G, and caspases-3, -8, and -9 according to a qPCR analysis. In a Western blot analysis, increases in levels of ATG5/12, beclin, p62, and LC3 conversion rates were found after bromelain treatment. Levels of cleaved caspase-3, caspase-8, caspase-9, and poly(ADP ribose) polymerase (PARP)-1 increased after bromelain exposure. This study explored the role of bromelain in CRC while giving insights into its mechanisms of action. This compound can offer a cheap alternative to current therapies.

## Introduction

Colorectal cancer (CRC) is one of the most prevalent and deadly tumor types worldwide [1]. When treatments with curative intent are not considered possible, patients are administered cytotoxic chemotherapy often combined with targeted therapy. Despite advances in cytotoxic and targeted therapies, the 5-year survival rate with metastatic disease is still a mere 12.5% [2]. The reason for treatment failure is thought to be acquired resistance to pharmacological therapy, which occurs in 90% of patients with metastatic cancer [3]. Resistance to pharmacological treatment remains the greatest obstacle in managing incurable metastatic CRC. Therefore, new effective or alternative therapies are urgently needed for CRC in the clinic.

Bromelain is an extract of pineapple and a kind of protease that has anti-inflammatory actions, fibrinolytic effects, anticancer activities, and immunomodulatory effects [4–6]. The human intestines can absorb bromelain without degradation or loss of its biological properties [7]. Several studies showed that bromelain can inhibit cell growth and induce cell apoptosis in different cancers through different pathways [8–10]. In gastric cancer, bromelain treatment reduced cell growth accompanied by significant DNA perturbation [11]. In glioblastomas, bromelain inhibited adhesion, migration, and invasion in primary cell lines, but had no effect on cell proliferation [12].

Romano et al. indicated that bromelain suppressed proliferation and induced apoptosis through activation of the extracellular signal-regulated kinase (ERK)/AKT pathway and reduced H_2_O_2_-induced reactive oxygen species (ROS) production [13]. A combination of bromelain and N-acetylcysteine produced increased inhibition of proliferation and survival of gastrointestinal (GI) cancer cells [14]. However, the effects of bromelain are still not completely understood. Our study attempted to elucidate the effects of bromelain on CRC progression. We found that bromelain could inhibit CRC progression *in vitro* and *in vivo* through induction of ROS production and activation of the autophagy pathway. These findings provide new information for therapeutic applications of bromelain in CRC.

## Materials and methods

### Chemicals, reagents, and cell culture

Bromelain and dimethyl sulfoxide (DMSO) were obtained from Sigma Chemical (St. Louis, MO, USA). The DLD-1 (CCL-221), HT-29 (HTB-38), and HCT116 (CCL-247) cell lines were obtained from American Type Culture Collection (ATCC, Rockville MD, USA), and all cell lines had been isolated from human colon adenocarcinomas. Cells were cultured in RPMI 1640 with 10% fetal bovine serum (FBS), penicillin (100 U/mL), and streptomycin (100 μg/mL) in an incubator (at 37°C with 5% CO_2_).

### Sulforhodamine B (SRB) colorimetric assay

Cells (2×10^4^) were seeded in 24-well plates and incubated overnight. Different concentrations of bromelain (0~90 μg/mL) or its control (distilled H_2_O) were then used to treat cells for 48 h. After the incubation period, cells were fixed with 10% trichloroacetic acid overnight and stained with protein-bound SRB for 30 min. Then, excess dye was removed by repeatedly washing the cells with 1% acetic acid. The dye was dissolved in a 10 mM Tris base solution for optical density (OD) value measurement at 515 nm on a microplate reader, based on the determination of the cellular protein content. The multiple of change was calculated as the OD value of cells treated with bromelain relative to the control group. Cells with control treatment were taken as the basal line group.

### Xenotransplantation

This assay was performed by the Taiwan Zebrafish Core Facility—Human Disease Model Resource Center (Miaoli, Taiwan). In brief, at 2 days post-fertilization (dpf), zebrafish embryos were dechorionated and subsequently anesthetized with tricaine (0.04 mg/ml, Sigma). HCT116 or DLD-1 cells were labeled with CM-Dil (red fluorescence) (Vybrant; Invitrogen, Carlsbad, CA, USA). Approximately 200 cells (4.6 nl) were implanted into the yolk of each 2-dpf embryo using a Nanoject II Auto-Nanoliter Injector (Drummond Scientific, Broomall, PA, USA). After injection, zebrafish embryos were washed once with fish water and incubated for 1 h at 28°C. The embryos were exposed to different amounts of bromelain (0~300 μg/ml), checked for fluorescent cells at 2 h post-transplantation and then examined at 1 and 3 days post-injection (dpi) by fluorescence microscopy. A comparison of the 1- and 3-dpi stages revealed the proliferative activity between the vehicle control and bromelain treatment.

### Ethics statement

All mouse experiments were performed in strict accordance with regulations of the Institutional Animal Care and Use Committee or Panel (IACUC/IACUP), and the protocol was approved by the IACUC, Taipei Medical University (LAC-2018-0198).

### Animal models for the therapeutic study

All procedures were carried out according to the *Animal Protection Act* (Act/APC) and the Experimental Animal Ethics Committee of the Council of Agriculture (CoA) of the Executive Yuan, Taiwan. Five-week-old male BALB/cAnN.Cg-Foxn1nu/CrlNarl (nude) mice were purchased from the National Laboratory Animal Center (Taipei, Taiwan). All mice were housed five to a cage in polycarbonate plastic cages with soft bedding in an air-conditioned room at a temperature of 23 ± 2°C and a humidity of 50% ± 10% with a 12-h light/dark cycle. Animals were allowed ad libitum access to an irradiation-sterilized commercial diet and sterilized water in plastic bottles. Mouse health was monitored by a veterinarian of the TMU-Animal Center.

HCT116 and HT-29 cells were suspended in phosphate-buffered saline (PBS) to a final cell density of 10^7^ cells/mL. A 0.1-mL volume of the cell suspension was subcutaneously (s.c.) injected into the left side of each mouse. When the mean tumor diameter reached 5 mm, mice were randomly separated into two groups (a vehicle group and a bromelain-treated group). Experimental mice were treated with an intraperitoneal dose of 20 mg bromelain/kg body weight (BW) twice a week. Control mice were given an equal volume of PBS. Tumor dimensions and body weights were recorded twice per week after bromelain administration. Tumor volumes were calculated using the equation (L×w^2^)/2, where L and w are the larger and smaller tumor dimensions, respectively [15]. All animals were euthanized under isoflurane inhalation anesthesia and cervical dislocation. To assess treatment-related toxicity, animals were also weighed, and all tumors were excised and weighed.

### Total ROS/superoxide detection utilizing the FlexiCyte protocol

HCT116 or HT-29 cells (2.4×10^5^) were seeded in six-well plates overnight and exposed to bromelain or the vehicle for 24 h. Cells were then harvested and stained with two fluorescent dyes from the ROS-ID Total ROS/Superoxide detection kit (ENZ-51010, Madison, NY, USA). The intensity of the green dye (total ROS detection reagent) represents the level of real-time oxidative stress. On the other hand, the orange dye (superoxide detection reagent) provides exclusive detection of superoxide in living cells. In addition, harvested cells were stained with Hoechst-33342, which labels nuclei and is used for detection of the total cell population. Cells were incubated at 37°C for 15 min in a heating block. The florescence intensity and number were detected and measured with the NucleoCounter NC-3000 system (ChemoMetec A/S, Allerod, Denmark).

### Autophagy and lysosome detection by the FlexiCyte protocol

HCT116 or HT-29 cells (2.4×10^5^) were seeded in six-well plates overnight and then incubated with bromelain or the vehicle for 24 h. Cells were harvested and stained with fluorescent dyes from the CYTO-ID Autophagy Detection Kit (NZ-51031). The green dye stained autophagic vacuoles, including pre-autophagosomes, autophagosomes, and autolysosomes, but with minimal staining of lysosomes. On the other hand, the LYSO-ID Green detection kit (ENZ-52405) was simultaneously applied to measure the level of lysosomes. In addition, harvested cells were stained with Hoechst-33342, which labels nuclei and is used for detecting the total cell population. Cells were incubated at 37°C for 15 min on a heating block. The florescence intensity and number were detected and measured using the NucleoCounter NC-3000 system (ChemoMetec A/S).

### Reverse-transcription polymerase chain reaction (RT-PCR) and quantitative RT-(q)PCR analyses

Total RNA was extracted using the TRIZOL reagent according to the manufacturer's instructions (Invitrogen Life Technologies, Carlsbad, CA, USA). Total RNA (8 μg) was used for the RT reactions in a 20-μl reaction volume to synthesize complementary cDNA using a cDNA Synthesis Kit (Invitrogen Life Technologies). For validation, a real-time RT-PCR was performed using the Power SYBR-Green real-time RT-PCR system and ABI 7500 FASTTM detection system (Applied Biosystems, Foster City, CA, USA). The RT-qPCR was performed using ABI SYBR Green Master Mix (Applied Biosystems). Thermal cycling was performed in an ABI 7500 FAST TM, and the qPCR conditions were as follows: 95°C for 10 min followed by 40 cycles of 95°C for 15 s and 60°C for 1 min. A melting curve was run after the PCR cycles, followed by a cooling step. Each sample was run in triplicate in each experiment, and each experiment was repeated three times. Expression levels of target genes were normalized to the expression level of GAPDH. The sequences of all primers are listed in **Table 1**.

**Table 1:** Primer sequences used for the qPCR analysis with target genes.

Name		Primer sequences
AIF-1	Forward	5'-GGGAGGACTACGGCAAAGGT-3'
	Reverse	5'-CTTCCTTGCTATTGGCATTCG-3'
Endo G	Forward	5'-GTACCAGGTCATCGGCAAGAA-3'
	Reverse	5'-CGTAGGTGCGGAGCTCAATT-3'
Caspase-3	Forward	5’-CAGTGGAGGCCGACTTCTTG-3’
	Reverse	5’-TGGCACAAAGCGACTGGAT-3’
Caspase-8	Forward	5'-GGATGGCCACTGTGAATAACTG-3'
	Reverse	5'-TCGAGGACATCGCTCTCTCA-3'
Caspase-9	Forward	5'-TGTCCTACTCTACTTTCCCAGGTTTT-3'
	Reverse	5’GTGAGCCCACTGCTCAAAGAT-3’

### Terminal deoxynucleotidyl transferase-mediated nick end labeling (TUNEL) assay

Cells were plated in six-well plates at 3×10^5^ cells/well overnight and then treated with bromelain or H2O as the vehicle control for 48 h. Cells were harvested and washed with PBS. The cellular DNA fragmentation morphology was detected by a TUNEL assay using an Apo-BrdU in situ DNA Fragmentation Assay Kit (Bio Vision, Mountain View, CA, USA) according to the manufacturer’s instructions. TUNEL-positive cells were then analyzed using fluorescence microscopy.

### Protein extraction and Western blot analysis

Cells were treated with bromelain or the vehicle for 48 h. Proteins were analyzed by Western blotting, as previously described [16]. In brief, proteins (20 μg) were separated by sodium dodecylsulfate polyacrylamide gel electrophoresis (SDS-PAGE), and electrotransferred onto polyvinylidene difluoride membranes (GE Healthcare Piscataway, NJ, USA). Membranes were incubated with ATG5, ATG12, Beclin-1, light chain 3 (LC3), cleaved (c)-caspase-3, c-caspase-8, c-caspase-9, or c-PARP antibodies at 4°C overnight, and subsequently probed with the respective secondary antibody. The products were visualized with an enhanced chemiluminescence reagent (GE Healthcare Piscataway, NJ, USA), and detected using VersaDoc 5000 (Bio-Rad Laboratories, Hercules, CA, USA).

### Statistical analysis

Data of the results are presented as the mean±standard deviation (SD), and were from at least triplicate experiments. Significant differences were analyzed using Student’s *t*-test (two-tailed) to compare two groups. A value of *p*<0.05 was considered statistically significant.

## Results

### Bromelain treatment suppresses the survival of CRC cells

**Fig 1:**
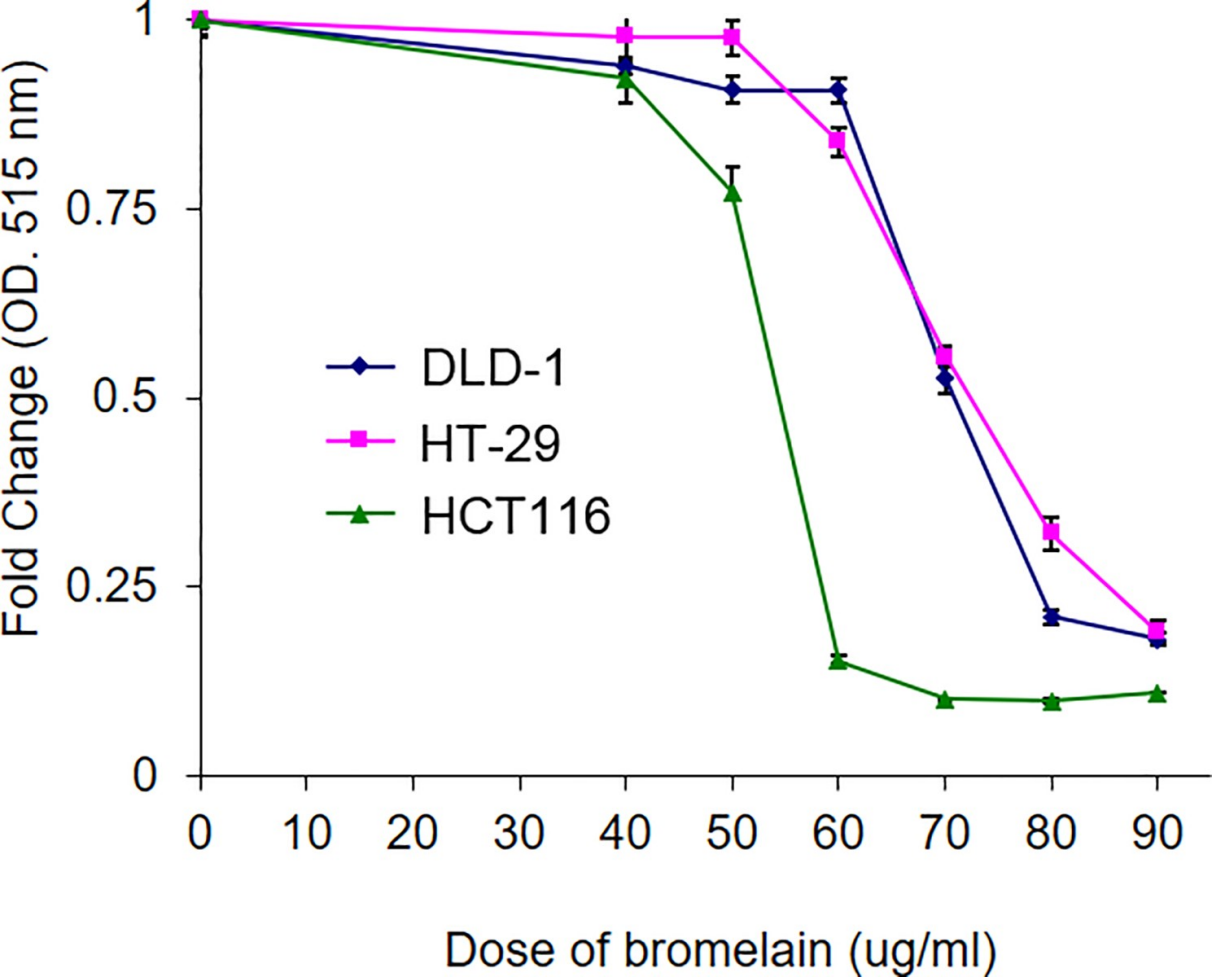
Bromelain treatment caused a reduction in the survival of colorectal cancer (CRC) cells. CRC cells (HT-29, HCT116, and DLD-1) were incubated with different doses of bromelain (0~90 μg/ml) for 48 h. Cell survival rates were determined by a sulforhodamine B assay. The relative vehicle survival rate was set to 100%. Dose-dependent cytotoxic effects of bromelain are presented in the graph. The IC50 value of bromelain was around 60~70 μg/ml with 48 h of treatment. Data are presented as the mean±SD of three independent experiments run in triplicate. ** *p*<0.01.

We tested the anticancer effects of bromelain in three different CRC cell lines (HT-29, HCT116, and DLD-1) using the SRB assay. CRC cells was seeded in 24-well plates and incubated with different concentrations (0~90 μg/ml) of bromelain for 48 h. The survival rate was determined by SRB. As shown in Fig 1, survival rates of CRC cells were reduced after bromelain treatment in a dose-dependent manner. The 50% inhibitory concentration (IC_50_) of bromelain was 50 μg/ml for HCT116 cells, and 70 μg/ml for HT-29 and DLD-1 cells. HCT116 cells were obviously more sensitive to bromelain treatment.

### Bromelain treatment suppresses proliferation in a zebrafish model

**Fig 2:**
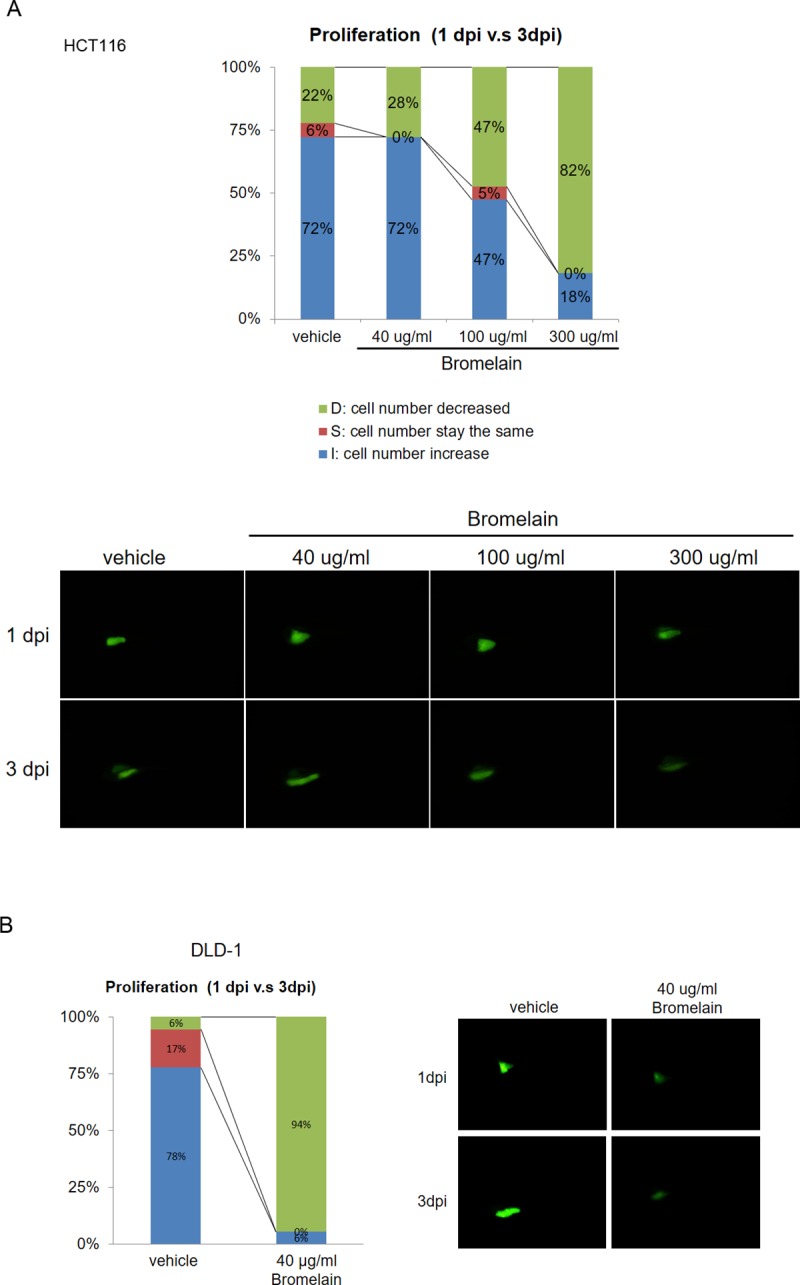
Bromelain treatment suppressed proliferation of HCT116 cells in a zebrafish model. The inhibitory effect of bromelain on colorectal cancer growth was confirmed using a xenotransplantation assay performed in a zebrafish system. (A) In HCT116 cells, the percentage of cells in embryos decreased after exposure to bromelain from 72% (vehicle) to 47% (100 μg/ml) and 18% (300 μg/ml). An increase in the percentage of cells in embryos was found from 22% (vehicle) to approximately 82% (bromelain). (B) In DLD-1 cells, an increase in the percentage of cells in embryo was found from 6% (vehicle) to approximately 94% (bromelain). The figure is representative of the fluorescence intensity in embryos. Data are presented as the mean±SD. ** *p*<0.001.

We further confirmed the inhibitory effect of bromelain with a xenotransplantation assay. Briefly, HCT116 cells were labeled with fluorescent dye and implanted into zebrafish embryo yolks. Different amounts (0~300 μg/ml) of bromelain were incubated with HCT116-implanted embryos. The fluorescence intensity was monitored at the 1- and 3-dpi stages. The proliferative activity of cells is indicated by the ratio of the comparison of the 1- vs. 3-dpi stages. As shown in Fig 2A, increases in the ratio of cell numbers in embryos were similar in vehicle-treated and the 40 μg/ml bromelain-treated group. However, with an increase in the bromelain concentration, the ratio dramatically decreased to 47% (100 μg/ml) and 18% (300 μg/ml). In DLD-1 cells, the cell number decrease population was increased after bromelain treatment (Fig 2B). These results indicated that bromelain can suppress CRC cell proliferation in a zebrafish model.

### Bromelain treatment suppresses CRC progression in a xenograft mouse model

**Fig 3:**
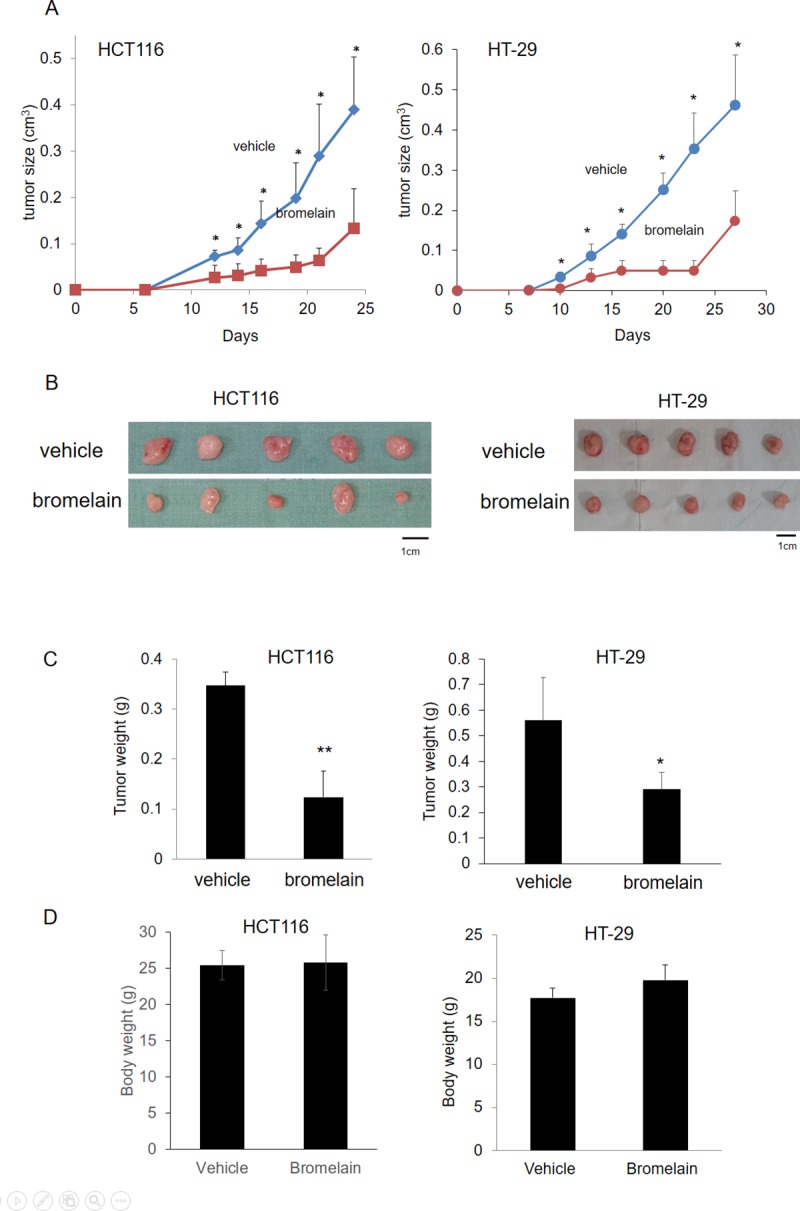
Bromelain treatment suppressed colorectal cancer (CRC) progression in a xenograft mouse model. (A) The growth rate was slower in the bromelain-treated group in both the HCT116 and HT-29 cell lines. (B, C) The tumor size and tumor weight were significantly reduced to 30% compared to the vehicle-treated group. (D) There was no significant difference in body weights between the vehicle- and bromelain-treated mouse groups. * *p*<0.05.

To further confirm our *in vitro* findings, a xenograft mouse model was used to evaluate the inhibitory effect of bromelain on CRC progression. In total, 10^6^ HCT116 or HT29 cells were injected into the left side flanks of nude mice. The tumor volume and body weight of the mice were recorded twice per week. The tumor growth rate was slower in the bromelain-treated group (Fig 3A). Tumor sizes and tumor weights were significantly reduced by 30% compared to those of the vehicle-treated group (Fig 3B and 3C). However, there was no significant difference in body weights between the vehicle- and bromelain-treated mice groups (Fig 3D). These results indicated that bromelain inhibited CRC progression in vivo.

### Bromelain treatment induces oxidative stress and superoxide production

**Fig 4:**
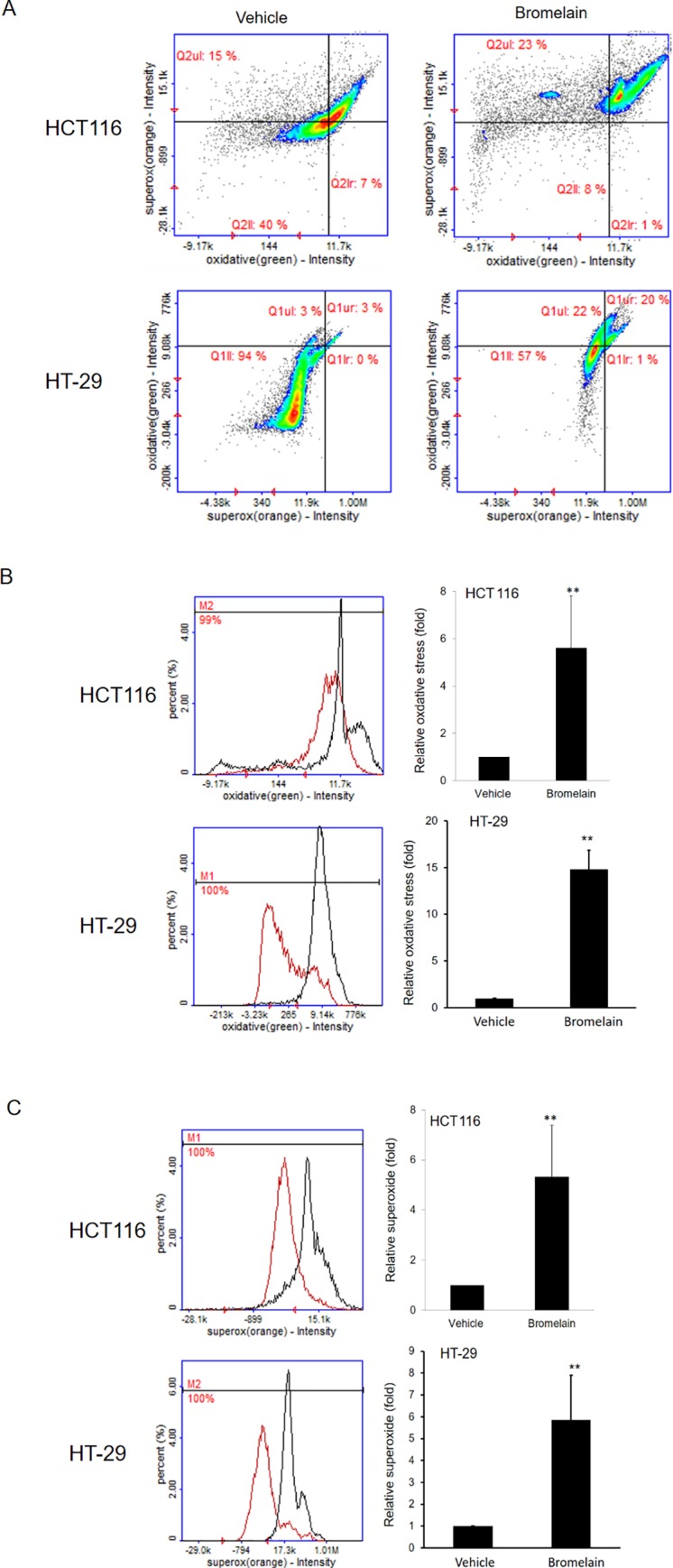
Bromelain treatment induced oxidative stress and superoxide production. (A) Both oxidative stress and superoxide production increased as indicated by the fluorescence intensity in HCT116 and HT-29 cells after bromelain exposure. (B) Oxidative stress increased 6-fold in bromelain-treated cells compared to the vehicle control in HCT116 and HT-29 cells. (C) Superoxide production increased after bromelain treatment in both HCT116 and HT-29 cells. Data are presented as the mean±SD of at least three independent experiments. ** *p*<0.005.

ROS production plays important roles in cancer progression and drug responses [17–19]. We further monitored ROS production in HCT116 and HT-29 cells after 50 μg/ml bromelain or vehicle treatment. The relative ROS production was calculated as the level of oxidative stress or superoxide induced by bromelain or vehicle treatment for 24 h (Fig 4A). As shown in Fig 4B, oxidative stress increased by 6-fold in bromelain-treated cells compared to vehicle control cells. Similar induction was found in levels of superoxide produced (Fig 4C). These results indicated that bromelain may induce the production of oxidative stress and superoxide.

### Increases in autophagy and lysosome formation after bromelain treatment

**Fig 5:**
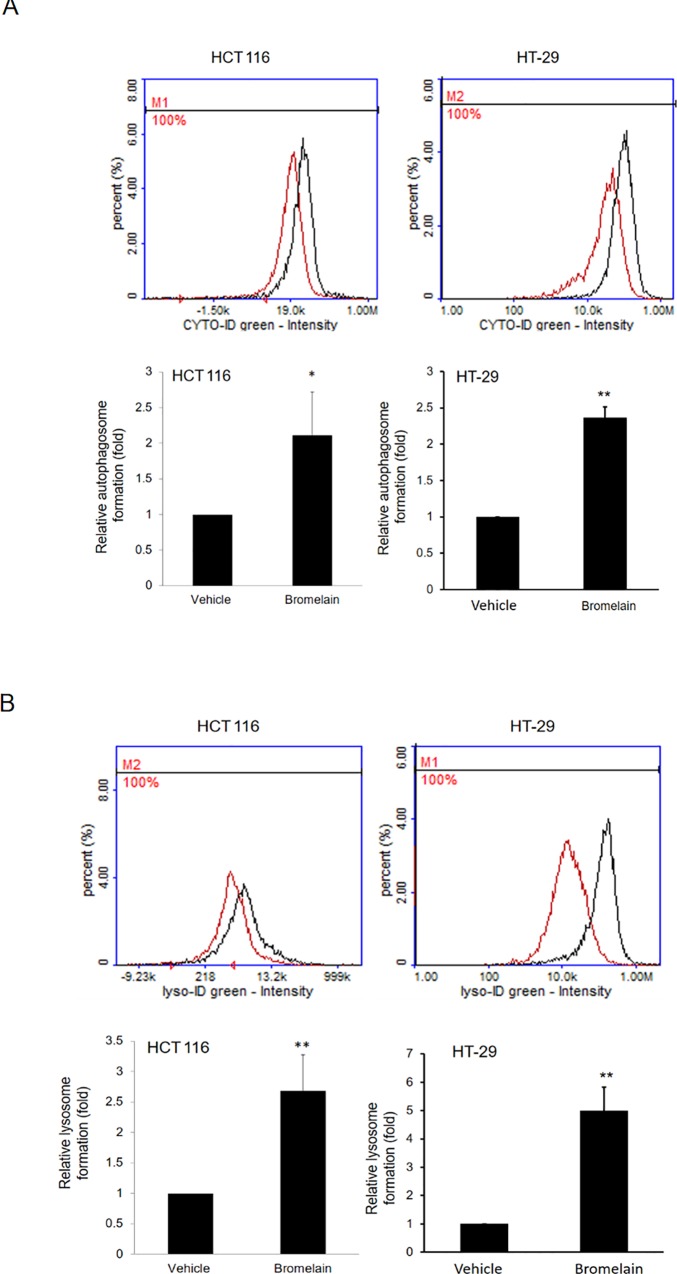
Autophagy and lysosome formation increased after bromelain treatment. (A) Autophagosome formation: Positive signals of autophagosomes increased up to 2-fold in bromelain-treated HCT116 and HT-29 cells compared to the vehicle control. (B) Lysosome formation: Similar results of induction of lysosome formation were found after bromelain treatment in HCT116 and HT-29 cells. Data are presented as the mean±SD of at least three independent experiments. * *p*<0.05; ** *p*<0.005.

Activation of autophagy or lysosomes may be the possible mechanism underlying the induction of apoptosis in cancer therapy [17,20,21]. We further examined autophagy activation and lysosome induction after exposure to bromelain for 24 h. Autophagy signals were determined by specific fluorescent dyes from the CYTO-ID Autophagy detection kit (NZ-51031). The green dye stained autophagic vacuoles. As shown in Fig 5A, positive signals of autophagosomes increased 2~3-fold in bromelain-treated cells. As to lysosome formation, similar induction results were found after bromelain treatment as measured by the LYSO-ID Green detection kit (ENZ-52405) (Fig 5B). These results indicated that bromelain treatment may cause activation of the autophagy pathway and lysosome formation.

### Bromelain exposure causes cell apoptosis

**Fig 6:**
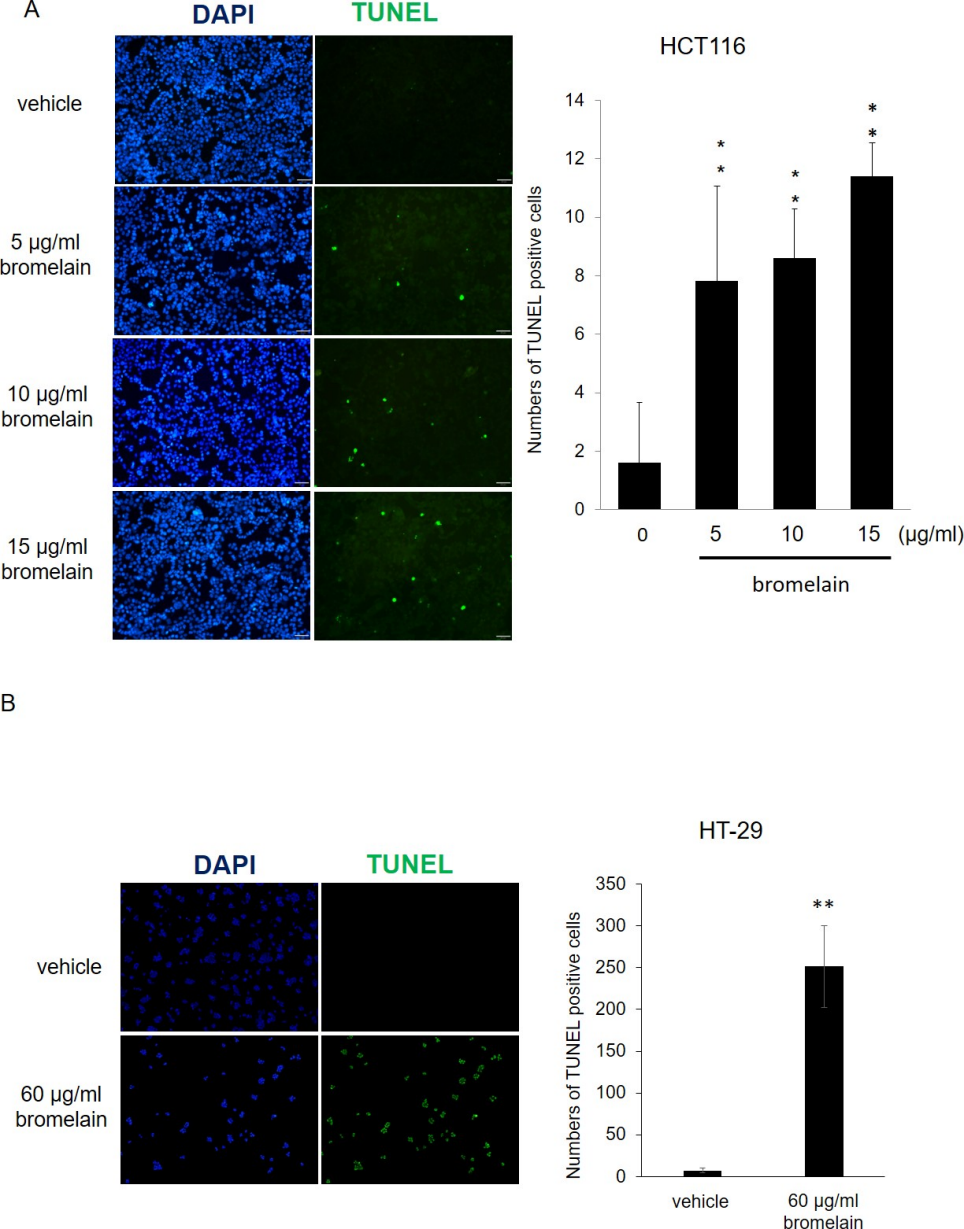
Bromelain treatment induced cell apoptosis as monitored by a TUNEL assay. (A) Representative fluorescent figure of the apoptotic assay. Blue DAPI was used to stain nuclei, and TdT tagged with a green fluorochrome was used to detect apoptotic DNA fragmentation. There were few cells with positive apoptotic signals in the vehicle control sample. However, exposure to different amounts (5~15 μg/ml) of bromelain induced dramatically more apoptotic signals in a dose-dependent manner.

To further confirm whether or not bromelain treatment can induce cell apoptosis, a TUNEL assay was applied. As shown in Fig 6, we found that there were few cells with positive signals in the vehicle control sample. However, after exposure to different concentrations (5~15 μg/ml) of bromelain, numbers of cells with positive signals dramatically increased in a dose-dependent manner. This indicated that bromelain inhibited cell proliferation through the induction of apoptosis.

### Bromelain treatment induces autophagy and apoptotic proteins

**Fig 7:**
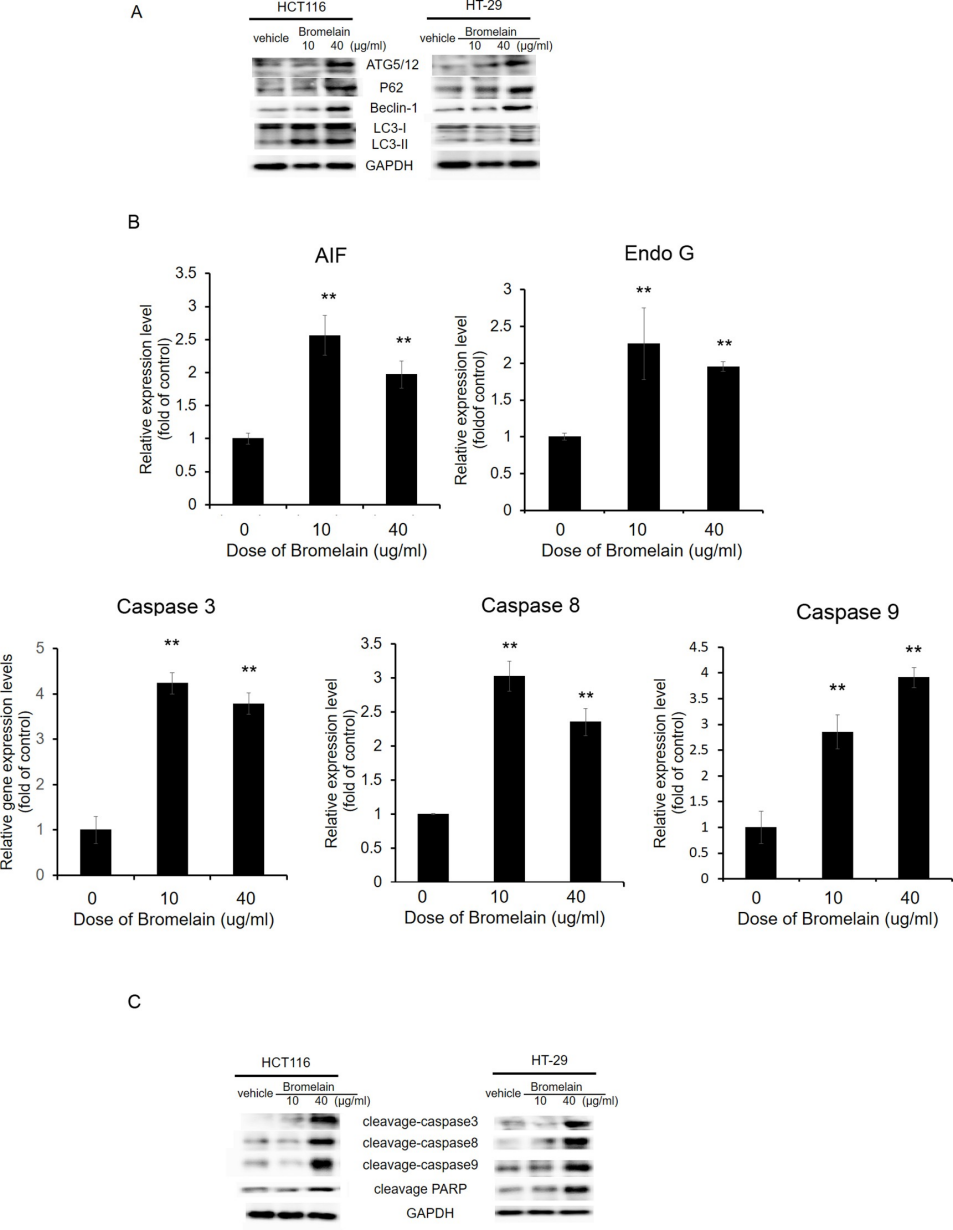
Bromelain treatment induces apoptosis. (A) Protein levels of autophagy-related molecules (ATG5/12, Beclin-1, LC3, and P62) in vehicle-treated and bromelain-treated HCT116 and HT-29 cells were detected by Western blotting. (B) A qPCR analysis of apoptotic gene expressions in HCT116 cells treated with 0, 10, and 40 μg/ml of bromelain. Levels of AIF, Endo G, caspase-3, caspase-8, caspase-9 dramatically increased after bromelain treatment. (C) Levels of cleaved (c)-PARP, c-caspase-3, c-caspase-8, and c-caspase-9 in vehicle-treated and bromelain-treated HCT116 and HT-29 cells were detected by Western blotting. Data are presented as the mean±SD of at least three independent experiments. * *p*<0.05; ** *p*<0.005.

To dissect bromelain's action mechanism, we further tested autophagy and apoptosis pathway-related genes by Western blotting and a qPCR. Levels of autophagy-related proteins, ATG5/12, beclin, p62, and LC3I/II, were determined, and increases in ATG5/12, beclin, and p62 levels were found after bromelain treatment (Fig 7A). In addition, the conversion of LC3-I to LC3-II increased after bromelain treatment. This is a reflection of bromelain-induced autophagy. As to apoptosis, we first checked the level of apoptosis-related biomarkers, including apoptosis-inducing factor (AIF), Endo G, and caspases-3, -8, and -9, in HCT116 cells. As shown in Fig 7B, expression levels of AIF, Endo G, and caspases-3, -8, and -9 dramatically increased after bromelain treatment in dose-dependent manners. The levels of cleaved caspase-3, caspase-8, caspase-9, and PARP significantly increased after bromelain treatment (Fig 7C). These results indicated that bromelain may suppress CRC proliferation through modulating autophagy or apoptosis-related gene expressions.

## Discussion

CRC is a leading cause of cancer deaths worldwide and has the potential to spread into the peritoneal cavity. Surgical excision has shown limited curative effectiveness in local regional cancer spread, and so this disease is commonly treated with cytotoxic chemotherapy [22]. Mucins form a relatively cell type-specific barrier from specialized epithelium. Evidence indicates that mucins secreted by the mucosa have been implicated in the pathogenesis of GI cancers, including CRC [23]. Pineapple has been used for centuries to treat alimentary diseases. The active compound of the pineapple stem is bromelain, and is primarily a proteolytic enzyme [24]. Gerard in 1972 and Goldstein in 1975 reported on the treatment efficacy of bromelain against malignant tumors [25,26]. However, those reports were considered to be anecdotal evidence. Bromelain has shown modulatory effects on wound debridement, inflammation, immune responses, and plate aggregation [9,24,27–29]. There are limited preclinical and clinical observations of bromelain as an anticancer substance. This study explored the role and mechanisms of action of bromelain in CRC.

Bromelain exhibited a cell-specific role as a survival regulator. In mouse cardiomyocytes, bromelain increased cell survival and decreased apoptosis, thus showing protection against ischemia-reperfusion injury [30]. In our study, we demonstrated that bromelain induces caspase-dependent apoptosis in CRC cells. In agreement with previous studies, bromelain showed proapoptotic effects by the upregulation of p53 accompanied by activation of caspase-mediated apoptosis [8,31,32]. Exposure to bromelain reduced cell proliferation and induced apoptosis via increasing expression levels of caspases-3, -8, and -9 (Fig 7), which is consistent with previous findings in GI malignancies [14]. In addition, we found elevated levels of apoptotic proteins like AIF and EndoG in CRC cells treated with bromelain. AIF and EndoG contribute to a caspase-independent pathway of apoptosis (positive intrinsic regulator of apoptosis) and then causes DNA fragmentation and chromatin condensation [33]. This means both caspase-dependent and caspase-independent apoptotic pathways were triggered in CRC cells treated with bromelain.

Tumor cells are vulnerable to excessive oxidative stress. Many natural compounds provide antitumor activity through modulating oxidative stress [17]. AIF is a key mediator in the respiratory chain and metabolic redox reactions in mitochondria [34]. We found that bromelain induced elevation of ROS and superoxide as shown in Fig 4. A critical event in developing GI tumors is damage to antioxidant defenses by oxidative stress in intestinal epithelial cells [35]. ROS can damage cell structures such as proteins and carbohydrates, and change their functions. Regulating the balance between reducing and oxidizing states is necessary for cell viability and proliferation. ROS possess both harmful effects that contribute to pathological conditions, including cancer, and beneficial effects that induce apoptotic processes in cancer cells [35]. Romano et al. also reported that bromelain inhibited ROS induced by hydrogen peroxide in a dose-dependent manner in CRC cells [13]. This implies that exposure to bromelain causes the overproduction of ROS and subsequent activation of apoptosis.

Autophagy is a key mechanism for cancer cells adapted to a strict environment including oxidative stress. Autophagy is involved in cancer progression and drug resistance [36]. Alleviation of ROS induced by hydrogen peroxide and preventing apoptosis through lysosome degradation can also be helped by the overexpression of the autophagy-associated protein, beclin-1 [37]. However, in cases where ROS are so excessive that autophagy induction is very high, as in our study, irreversible cell damage occurs, leading to apoptosis [38]. In addition, activation of autophagy was found to regulate caspase-dependent and -independent cell death by various signaling pathways [39]. Other studies also demonstrated that bromelain increases expressions of autophagy-related proteins including the autophagosome marker, LC3-II [14,40]. Together, bromelain treatment may activate autophagosome and lysosome formation and then induce apoptosis of CRC cells. Autophagy contributes to the second type of programmed cell death. Once autophagy is initiated, the autophagy-inducible beclin-1 complex contributes to phagophore formation and activation of downstream autophagic signals [41]. In addition to beclin-1, multiple autophagy-related proteins (ATGs) are required for LC3-associated phagocytosis, including all crucial components of the LC3 conjugation constituents (ATG5, ATG12, etc.). During autophagy, isolated cargo material is delivered with autophagosomes into the lysosomal system [42]. Autophagy-related proteins (ATGs) mediate bulk degradation of cytosolic material, and the lipidation of LC3 (LC3-II) is a key element in the capture of the autophagic cargo and autophagosomal membrane stabilization [43]. Lipidated LC3 enables the docking of specific cargos and adaptor proteins such as p62 (sequestosome-1). p62/sequestosome-1 is an ubiquitin-binding protein encoded by the SQSTM1 gene, and is an autophagosome cargo protein that targets other proteins which bind to it for selective autophagy [44–46]. Elevation of levels of ATG5/12, beclin, p62, and LC3 conversion are the most reliable marker of autophagy [40,47,48]. Our results showed that bromelain treatment activated both apoptotic and autophagic cell death in CRC cells.

Previous studies showed the antiproliferative effects of bromelain in different cancer cell lines, including glioblastoma cells, gastric carcinoma cells, ovarian cancer cells, breast cancer cells, and human epidermoid carcinoma cells [6,9]. Elevation of p53 and Bax, the decrease in Bcl-2, activation of caspases-3 and -9, and decreases in AKT/pAKT and mitogen-activated protein kinase (MAPK) signals were investigated in mouse skin tumors [8,32,49]. Furthermore, promotion of the cell cycle is modulated by cyclins and cyclin-dependent kinases (CDKs) [50]. Amini et al. demonstrated that bromelain exposure caused G_1_ arrest through decreasing cyclins D, A, and B in GI cancer [14]. The mucin, MUC1, is highly correlated with cell survival, invasion, and the metastatic ability of tumor cells [51]. MUC1 is overexpressed in 900,000 of the 1.4 million malignancies each year in the US [52,53]. Bromelain also exhibited anticancer functions by eliminating the oncoprotein, MUC1. In patients with inflammatory bowel disease, bromelain was shown to decrease secretion of inflammatory cytokines and chemokines. Emerging evidence also suggests that bromelain modulates nuclear factor (NF)-κB and cyclooxygenase (COX)-2, a key regulator of inflammation and GI malignancies [32,54,55]. The fibrinolytic, antiplatelet, and antithrombotic effects of bromelain were also recognized [56,57]. Bromelain showed benefits in controlling the chronic inflammatory microenvironment caused by malignancies, and enhanced immunity by ‘‘un-coating” cancer cells facing a host's defense [58,59]. Relevant to our present investigation, bromelain was found to be associated with activation of ROS-related apoptosis and autophagy, and inhibition of prosurvival pathways in CRC cells both in vitro, and in a zebrafish model and a xenograft mouse model.

In conclusion, we revealed that bromelain exhibits antiproliferative actions both *in vitro* and *in vivo*. Treatment with bromelain inhibited the proliferation of CRC cells through activating both caspase-dependent and -independent apoptosis and inducing programmed autophagic cell death. The role of bromelain in CRC treatment could be therapeutic and of economic importance and needs further investigation.
